# Perceptual Representation of Own Hand Size in Early Childhood and Adulthood

**DOI:** 10.1038/s41598-020-62206-5

**Published:** 2020-03-25

**Authors:** Serena Giurgola, Nadia Bolognini, Elena Nava

**Affiliations:** 1 Department of Medicine and Surgery, Ph.D. Program in Neuroscience, University of Milano-Bicocca, Milan, Italy; 2 Department of Psychology & Milan Center for Neuroscience (NeuroMI), University of Milano-Bicocca, Milan, Italy; 3 IRCCS Istituto Auxologico Italiano, Laboratory of Neuropsychology, Milan, Italy

**Keywords:** Psychology, Human behaviour

## Abstract

Hand size perceptual distortions characterize adult human cognition. Notwithstanding the importance of uncovering how hand size representation develops in humans, studies in this field are still at a preliminary stage. Indeed, it is yet to be understood whether hand size distortions are present and reliable in early childhood and whether they differ from adults’ distortions, offering a more in-depth insight into the emergence and development of such representations. We addressed this issue by comparing 4- to 6- year-old children and adults’ representation of their own hand size, as assessed with a 2-forced choice visual perceptual task. To test participants’ ability to estimate their own hand size, children and adults judged whether pictures of their own hand, resized to appear smaller or bigger than their own hand, matched or not its actual dimension. Results show that children aged 4 to 6 years tend to underestimate their own hand size, while adults underestimate their own hand more weakly. This evidence suggests that body-parts perceptual distortions are already in place in early childhood, and thus represent a characteristic of the human body representation.

## Introduction

In the past decade, a growing number of studies has shown that the knowledge of our own body is multidimensional, thus suggesting the existence of several types of conscious and unconscious body representations (i.e., various abstract representations of one’s own body^1–4^), including the knowledge of body-parts size. In this regard, neuropsychological evidence has shown that the perceptual representation of the sizes of the body and its parts are dramatically distorted in healthy human adults. In particular, there are systematic distortions of the relative size of body-parts, which resemble the distortions featuring the somatosensory homunculus of the primary somatosensory cortex (S1)^5,6^. Longo and colleagues^7^ postulated a cognitive model to explain the metric properties of body-parts, which contributes to the localization of the body in external space, the so-called ‘body model’. Such model assumes the existence of an implicit, stored, model of the body and its parts, that informs the position sense about the body’s metric properties, such as size and shape of each body-part. This proposal obtained empirical support by a series of experiments by Longo and co-workers^8^, who developed a ‘psycho-morphometric’ task to assess the representation of the hand dorsum underlying hand position sense (i.e. the ability of perceiving the spatial location of the own limbs without seeing them^9^); in healthy adults. Specifically, by using a pointing task, Longo *et al*.^8^ extracted the configuration of the subject’s judgments regarding the felt location of hand landmarks (5 on the tips and 5 on the knuckles, respectively), hence reproducing an implicit map of the perceived hand shape and dimension. This resulted in a distorted perceptual representation of own hand structure, characterized by overestimated hand width and underestimated finger length. Instead, Linkenauger and co-workers^5^, by employing a visual estimation task to examine the perceptual distortions of body-parts size, have found remarkable distortions in the perceived size of body proportions. In particular, perceptual distortions occurred when subjects compared the length of one body-part to another, but not when estimated the size of a body-part with respect to a noncorporeal object, or when judging noncorporeal objects of the same size as their own body-parts. Of interest, the perceived length of the body-parts varied accordingly to their tactile acuity, hence reproducing the S1 homunculus: body-parts with lower tactile acuity were perceived as enlarged (size overestimation) in comparison to those with higher tactile sensibility. This effect seems to represent a sort of compensation by which the perceptual system distorts the body part’s size to a magnitude that compensates for its differences in S1 maps^5^.

Overall, current evidence indicates that distortions of body-parts representation constitute an intrinsic feature of adult cognition^8,10^. However, the question remains as to whether perceptual distortions in body-parts size and shape arise during the developmental course or are only a characteristic of the adult body metric cognition. In this regard, only very few studies have directly assessed perceptual bodily distortions in children. For example, Le Cornu Knight *et al*.^11^ investigated tactile distance estimation (i.e., Weber’s law) in children aged between 5 and 7 years; children were asked to adjust the distance between two tactile stimuli presented to the left forearm and hand. Results showed that children, just as adults, perceived tactile distances as smaller when the two stimuli were presented within the boundaries of a single body-part (i.e., stimuli presented on the arm or on the hand) and they perceived the two stimuli as spaced farther apart when they were presented on two different body-parts (e.g., on the wrist and on the hand). The authors suggested that this stability in body distortions could be due to the fact that, while single body-parts change in size and proportion (given that children’s bodies rapidly grow across development), the relationship between the proportions of the different body-part, as well as their relative location, remain quite stable. On the other hand, children overestimated tactile distances, while adults commonly tend to underestimate them^12^.

Other developmental studies conducted in younger children showed that, by 30 months of age, toddlers possess a rudimentary topographic representation of their own body shape, structure and size^13^. In the study by Brownell and colleagues^13^, toddlers were administered five different tasks assessing their body topography and size; however, the task employed covered the toddlers’ awareness of own body representation, rather than directly measuring potential body distortions. For example, in one of the tasks toddlers were invited to put on doll clothes that were clearly too small for them to wear. The attempts of toddlers to actually put the clothes on were taken as evidence of immaturity of their body representation.

The issue of the development of body representation has been the focus of recent studies conducted in 4–5- year-old children, which took advantage of multisensory bodily illusions.

By means of the Rubber Hand Illusion (RHI), several studies have shown that while the sense of body ownership, i.e., a body sub-representation pertaining the feeling that body-parts belong to ourselves^14^, is already adult-like by age of 4 years^15,16^, the sense of body position still develops up to 10 years of age^17^. Intriguingly, a recent study examined the relationship between body size changes and corresponding updates in body representation, investigating to what extent children are able to update their own body representation in order to match a changed body-part size^18^. In the study by Filippetti *et al*.^18^, 6- to 8- year-old children were exposed to the RHI, while watching either a regular (child-like) or a bigger (adult-like) size fake hand being stroked in a synchronous or asynchronous fashion with their own hand. The synchronous (but not the asynchronous) stroking with both the regular and the bigger rubber hand modulated the sense of body ownership, showing that children reported owning the external fake hand regardless of its size. Thus, visuo-tactile inputs influenced body ownership in 6- to 8- years-old children, regardless of the perceived variations of the body metric properties. Interestingly, this result mimics the pattern observed in adults too. Indeed, Pavani and co-workers^19^, demonstrated that it is possible to induce illusory ownership of a fake hand regardless of its size (that could be smaller or bigger than the size of a normal hand of an adult person), at least as detected by subjective measures (i.e. questionnaires). In the same vein, variations of reach-to-grasp movements have been reported during object reaching and grasping as influenced by illusory distortions of the agent’s hand size^20,21^.

To date, there is only study that has addressed body distortions in children: Cardinali and colleagues^22^ have investigated body distortions in children aged between 6 and 10 years, by asking children to compare the 3D hand models of different sizes to their own hand. The most important finding was that children of the tested ages underestimated the size of their own hand, and, interestingly, this distortion increased with age. Notably, the distortions were selective for the body, as they did not extend to object size estimation, which was accurately estimated. The study by Cardinali *et al*.^22^ provides a first and very important insight into the development of bodily size distortions in childhood, but it also has left behind a series of unsolved issues.

The present study aims at further exploring the development of body size perception, firstly by testing children aged between 4 and 6 years of age, to verify whether body-parts size distortions at a perceptual level gradually develop in early childhood, as shown for other body representations^17,23^. Importantly, we directly compared body-parts size distortions in children with those exhibited by adults; this comparison is essential for revealing whether bodily size representation undergoes a developmental change, or it is already adult-like in younger childhood. Finally, perceptual distortions of the hand size in children were measured by using a computerized 2-forced-choice visual perceptual task, which presented pictures of the child’s hand resized to be smaller or bigger of the real ones. This paradigm, that we called Hand Size Task (HST), has been effectively used to track changes in hand size representation induced by S1 stimulation in adults^24^. At variance of the use of 3D hand models, as in Cardinali *et al*. ’s study, the HST has the advantage of exposing children to pictures of their real hand, and the child is presented with an equal number of rescaled pictures in which the size of the own hand is systematically increased and decreased. A Bayesian approach was then used to detect and compare perceptual hand size distortions in children and adults, also considering the ability of children and adults to recognize the veridical size of their own hand.

## Data Analysis

Statistical analyses were performed using JASP Software^25,26^. In general, the Bayesian framework offers a series of advantages in comparison to the more standard frequentist approach. First of all, it compares the predictive adequacy of two competing statistical models and provides a redistribution of probability between these competing accounts^27^. Second, it capitalises on prior knowledge to construct a more informative test.

As specified in the equation below, the prior model odds (i.e., the relative plausibility of the two hypotheses before seeing the data) multiplied by the Bayes Factor (i.e., the relative predictive performance for the observed data^28^) is equal to the posterior model odds, which is the relative plausibility of H_1_ and H_0_ after having seen the data.

$$\mathop{\overbrace{\frac{P({H}_{0}|Data)}{P({H}_{1}|Data)}}}\limits^{{\rm{Posterior}}\,{\rm{Odds}}}=\mathop{\overbrace{\frac{P(Data|{H}_{0})}{P(Data|{H}_{1})}}}\limits^{{\rm{Bayes}}\,{\rm{Factor}}}\times \mathop{\overbrace{\frac{P({H}_{0})}{P({H}_{1})}}}\limits^{{\rm{Prior}}\,{\rm{Odds}}}$$

The predictive updating factor that quantifies the change in beliefs about H_1_ and H_0_ based on observed data is known as the Bayes Factor. As shown in Table 1, BF_10_ indicates the Bayes Factor in favor of H_1_ over H_0_(whereas BF_01_ indicates the Bayes Factor in favor of H_0_ over H_1_): larger values of BF_10_ indicate stronger evidence in favor of H_1_, and a Bayes Factor of 1 commonly indicates that both hypotheses predict the data equally well.

**Table 1:** A descriptive and approximate classification scheme for the interpretation of Bayes factors BF_10_ (Lee & Wagenmakers, 2013; adjusted from Jeffreys, 1961).

Bayes factor Evidence category	
>100	Extreme evidence for H_1_
30–100	Very strong evidence for H_1_
10–30	Strong evidence for H_1_
3–10	Moderate evidence for H_1_
1–3	Anecdotal evidence for H_1_
1	No evidence
1/3–1	Anecdotal evidence for H_0_
1/10–1/3	Moderate evidence for H_0_
1/30–1/10	Strong evidence for H_0_
1/100–1/30	Very strong evidence for H_0_
<1/100	Extreme evidence for H_0_

As for parameter estimation, in Bayesian testing the interest centers on the posterior distribution of the model parameters, which reflects the relative plausibility of the parameter values after prior knowledge has been updated by the data. The estimation procedure starts by assigning a prior distribution (i.e., with unknown data). The information gathered with the data is then used to update the prior to the posterior distribution.

In our data, we used the Cauchy prior width set by default in JASP, which is centered around zero and has a width parameter of 0.707.

## Results

The experimental task was a 2-forced-choice visual perceptual task («Same»/«Different» response), developed in order to assess the perceptual estimation of the participants’ own hand size. Statistical analysis were conducted on the percentage of «Same» responses only; please note that the proportion of «Different» responses represents a complementary index (i.e., the subtraction of «Same» responses from the total accuracy score). In particular, for each participant we calculated the mean proportion of «Same» responses provided for each of the 11 hand sizes (from −15% to +15%). To detect differences between groups (children vs. adults), we performed a series of Bayesian Independent samples t-tests comparing the «Same» responses for each hand size. Furthermore, to document whether children and adults presented distortions of the perceived hand size, for both groups we made a series of Bayesian Paired samples t-tests comparing the «Same» Responses for each hand size (i.e., smaller and bigger, different size trials: from −15% to +15%) against the «Same» responses given when the size matched the real size of the participants’ hand (i.e., same size trials, 0% variations). If participants underestimate their own hand size, this would result in evidence in favor of H_0_, i.e., absence of difference between «Same» responses provided when the seen hand was smaller than «Same» responses provided when the seen hand was the real size of the participant’s. Similarly, if participants overestimate their own hand size, this would result in evidence in favor of H_0_, i.e., absence of difference between «Same» responses provided when the seen hand was larger than «Same» responses provided when the seen hand was the real size of the participant's.

On the contrary, if participants can discriminate between seen hand sizes and their own hand size, this would result in evidence in favor of H_1_.

### Hand size perception in children

**Figure 1:**
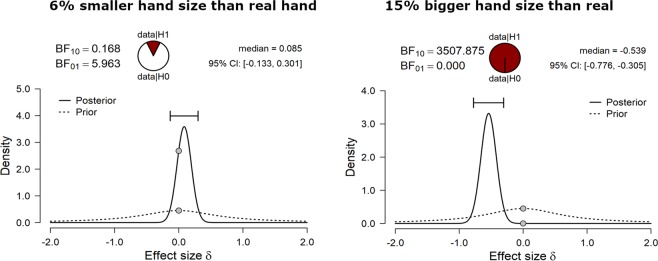
Example of prior and posterior distribution for the comparison between the proportion of «Same» responses in children, when the hand was 6% smaller (left panel) or 15% bigger (right panel) than their real hand.

To observe whether children underestimate or overestimate their hand size, we conducted a series of Bayesian Paired samples t-tests between the proportion of «Same» responses for the 10 different hand sizes (i.e. 5 smaller and 5 bigger) and the real hand size (i.e., 0% variations). The analyses revealed that children tended to underestimate the size of their own hand: indeed, there was evidence in favor of H_0_ particularly when the hand was −15% (BF_10_ = 0.23), −12% (BF_10_ = 0.17), and −6% (BF_10_ = 0.17, see Fig. 1, left panel, and Table 2).

**Table 2:** Bayesian Paired samples t-tests comparing percentage of «Same» responses for the ten different hand sizes (i.e., five smaller and five bigger than the real hand, respectively) to the real hand size (i.e., 0% variations) in children.

Bayesian paired samples t-tests			
Hand Size	Real Hand	BF_10_	error %
**−15**	**0**	**0.231**	**5.848e -6**
**−12**	**0**	**0.168**	**1.008–5**
−9	0	0.340	3.059e **-**6
**−6**	**0**	**0.168**	**1.007e -5**
−3	0	1.540	1.150e **-**6
**+3**	**0**	**0.125**	**1.625e -5**
+6	0	0.611	1.674e **-**6
**+9**	**0**	**13.904**	**3.471e -7**
**+12**	**0**	**12.082**	**3.815e -7**
**+15**	**0**	**3507.875**	**2.836e -9**

Moderate and strong evidence for either H_1_ or H_0_ are highlighted in bold.

On the contrary, there was strong evidence in favor of H_1_ when the children compared the larger hands to their own. That is, they discriminated very well when the hand was larger than their own, particularly when it was 9% (BF_10_ = 13.90), 12% (BF_10_ = 12.08), and 15% bigger than the children’ hand (BF_10_ = 3507.86, see example of the distribution in Fig. 1, right panel). However, it should be mentioned that children also reported their hand to be of the same size of the seen hand when this was +3% larger than their own (BF_10_ = 0.12), thus showing a slight tendency to overestimate.

Finally, to observe whether perceptual hand size distortion changes across ages 4 and 6, we run Bayesian regression analyses on each hand size with Age as predictor. As shown in Table 3, we mostly found anecdotal evidence in favor of H_0_ both for smaller and larger hand sizes. However, we found very strong evidence in favor of H_0_ when the hand was −6% (BF_10_ = 0.03) smaller than children’s own hand, and a moderate evidence in favor of H_0_ when the hand was +12% (BF_10_ = 0.29) and +15% (BF_10_ = 0.25) larger than their own. Overall, although not fully conclusive, there is some evidence suggesting that between ages 4 and 6 years, the perceptual representation of hand size remains quite stable.

**Table 3:** Bayesian regression analyses on each hand size with Age as predictor, for children.

Hand size	P(M)	P(M | data)	BF_10_	R^2^
−15 smaller than real hand	0.500	0.606	1.000	0.055
−12 smaller than real hand	0.500	0.872	1.000	0.095
−9 smaller than real hand	0.500	0.298	0.424	0.018
−6 smaller than real hand	0.500	0.202	0.025	0.002
−3 smaller than real hand	0.500	0.607	1.000	0.055
+3 bigger than real hand	0.500	0.808	1.000	0.082
+6 bigger than real hand	0.500	0.452	0.823	0.037
+9 bigger than real hand	0.500	0.250	0.333	0.011
+12 bigger than real hand	0.500	0.228	0.295	0.007
+15 bigger than real hand	0.500	0.197	0.246	0.001

### Hand size perception in adults

As for children, the proportion of «Same» responses for the different hand sizes was separately analyzed for adults, by comparing the 5 smaller and 5 bigger different hand sizes to the real hand size (i.e., 0% variations). The Bayesian Paired samples t-tests are reported in Table 4 and show that adults underestimated their hand size when the seen hand was even −15% smaller than their own (BF_10_ = 0.12) and, in a weaker way, when it was −12% smaller (BF_10_ = 0.35), but they were able to discriminate their own hand size when the seen hand size was −9% (BF_10_ = 5.51) and −6% (BF_10_ = 8) smaller than their real hand.

**Table 4:** Bayesian Paired samples t-tests comparing percentage of «Same» responses for the ten different hand sizes (i.e., five smaller and five bigger than the real hand, respectively) to the real hand size (i.e., 0% variations) in adults.

Bayesian paired samples t-tests			
Hand Size	Real Hand	BF_10_	error %
**−15**	**0**	**0.125**	**1.625e -5**
−12	0	0.349	2.925–6
**−9**	**0**	**5.514**	**6.205e -7**
**−6**	**0**	**8.000**	**4.975e -7**
−3	0	2.753	8.937e **-**7
**+3**	**0**	**1636.775**	**6.036e -9**
**+6**	**0**	**919439.641**	**5.541e -12**
**+9**	**0**	**1.720e** + **6**	**4.319e -9**
**+12**	**0**	**3.467e** + **7**	**2.811e -14**
**+15**	**0**	**6.188e** + **8**	**7.161e -16**

Moderate and strong evidence for either H1 or H0 are highlighted in bold.

**Figure 2:**
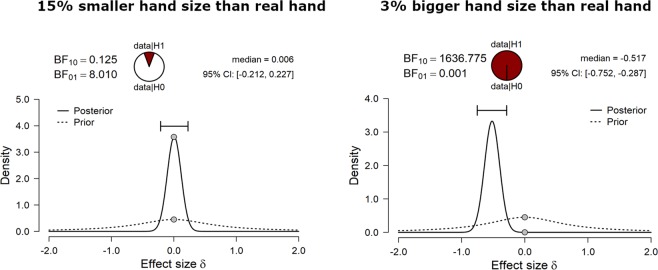
Example of prior and posterior distribution for the comparison between the proportion of «Same» responses in adults, when the hand was 15% smaller (left panel) or 3% bigger (right panel) than their real hand.

A strong evidence in favor of H_1_ was found when the seen hand was larger than the real hand size of the participant. Indeed, as in children, adults reported more «Same» responses when the hand was +3%, +6%, +9, +12 and +15 larger than their own (all BF_10_ > 1636, see Fig. 2 for an example of the distribution, and Table 4).

### Comparison between children and adults’ hand size perception

**Figure 3:**
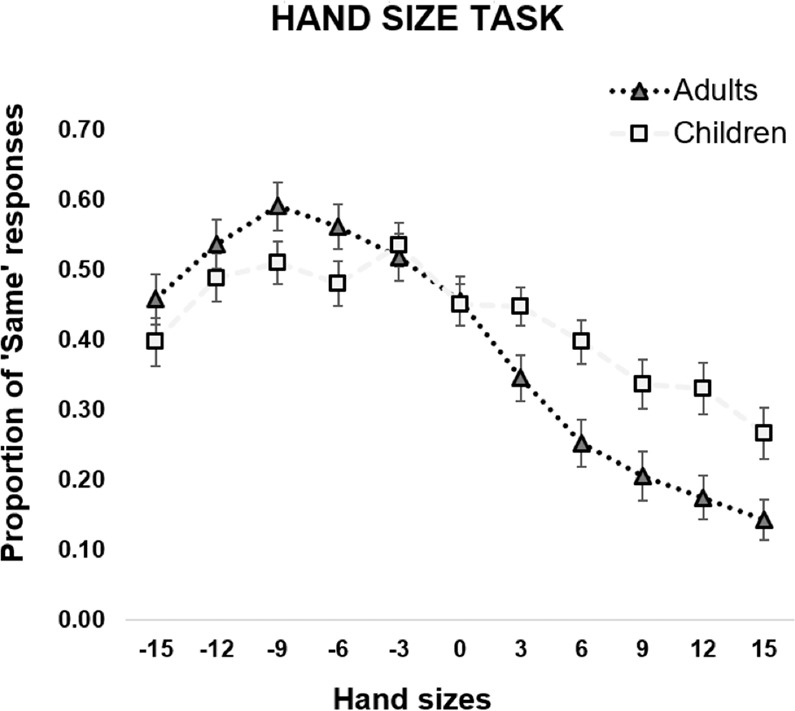
HST. Hand size perception for children and adults. X axis = percentage of the hand size change with respect to the participant’s own hand; negative values correspond to a reduction in hand size and positive values to an increase. Y axis = percentage of «Same» responses for each hand size. Grey line = children’s performance; black line = adults’ performance. Error bars = SEM.

Finally, to observe whether the perceptual hand distortions change between early childhood and adulthood, we run Bayesian Independent samples t-tests between «Same» responses reported by children and adults. The analysis showed that the proportion of «Same» responses of children and adults mostly differed when the size of the viewed hand was bigger than the participant's own hand (see Fig. 3). Indeed, children reported more «Same» responses than adults when the seen hand was 6% (BF_10_ = 12.76), 9% (BF_10_ = 4.21), 12% (BF_10_ = 19.47) and 15% (BF_10_ = 3.94) bigger than their own hand (see Table 5). On the contrary, when the viewed hand was smaller than the participant’s size, there was moderate evidence in favor of H_0_, that is, adults and children reported similar «Same» responses, particularly when the hand was −12% (BF_10_ = 0.27) and −3% (BF_10_ = 0.18) smaller than their own. A weaker evidence in favor of H_0_ was also observed when the seen hand was −15% smaller than the participants’ real one (BF_10_ = 0.35).

**Table 5:** Bayesian Independent samples t-tests comparing percentage of «Same» responses for the ten different hand sizes (i.e., five smaller and five bigger than the real hand, respectively) to the real hand size (i.e., 0% variations) between children and adults.

Bayesian Independent samples t-tests		
Hand Size	BF_10_	error %
−15	0.351	4.075e **-**6
**−12**	**0.274**	**5.2423–6**
−9	0.767	1.646e **-**6
−6	0.809	1.527e **-**6
**−3**	**0.182**	**7.6893–6**
**0**	**0.174**	**8.015e -6**
+3	2.294	3.299e **-**7
**+6**	**12.758**	**4.080e -9**
**+9**	**4.215**	**1.046e -7**
**+12**	**19.473**	**4.473e -9**
**+15**	**3.941**	**1.203e -7**

Moderate and strong evidence for either H_1_ or H_0_ are highlighted in bold.

When the viewed hand was of the same size of that of the participant (i.e., same size trials, 0% variations) the difference in «Same» responses between the two groups was similar (BF_10_ = 0.17). This last finding shows the absence of differences between adults and children in hand size estimation when their own hand, at its veridical size, was judged; this means that children and adults present a similar ability to recognize the actual size of their own hand, and the differences observed across groups result from a ‘genuine’ distorted estimation of different hand sizes.

## Discussion

The present study shows that both children aged 4–6 years, and adults, tend to underestimate the perceptual size of their own hand. Indeed, results from our HST show that when asked to recognize the size of their own hand among pictures of hands of different sizes, children and adults tend to recognize the viewed hand as same in size to their real hand when it is smaller than their real one, but not when it is larger. In particular, when misperception of own hand size was explored within each age group, we found that the tendency to underestimate one’s own hand size was more evident in children than in adults, as the latter were able to discriminate their own hand size when it was −6% and −9% smaller than their own. On the contrary, children did not reliably judge their hand size even when there was a small difference in the reduced size of the viewed hand.

Moreover, by comparing the responses of adults and children, no developmental change emerged from this comparison, hence children and adults had a comparable performance. We also did not find evidence of a developmental change in the regression analyses in which we compared the responses of the different children.

It is also noteworthy that children’s ability to visually recognize the real size of their hand (i.e., when the size of the seen hand matched their real hand size) was comparable to that of adults, supporting the reliability of the HST in children. In other words, one could claim that the differences observed between age groups could emerge as a consequence of a different perception of real hand size. However, the absence of difference suggests that the differences in distortion between children and adults cannot be ascribed to task difficulty but reflect a genuine misperception of hand size.

Our findings should be discussed in light of the study conducted by Cardinali *et al*.^22^. Indeed, they found that underestimation of hand size increases between age 6 and 10. How can our findings – of absence of developmental change across children and adults - be reconciled with Cardinali *et al*.^22^? In general, it could be that the children’s brain is not capable of updating the changes in body-parts growth, and as a result, always “keeps behind” and sees the own body-parts smaller than their actual dimensions.

Furthermore, the present findings in adults suggest that this “updating” mechanisms may apply to the adult brain too: because adults do not experience changes in body growth, it is reasonable that their perceptual distortions are not as strong as in children.

In support to our results, Gardner *et al*.^29^ investigated the perceived body size in children between 6 and 14 years of age, using videos depicting the life-size frontal image of the child, who was asked to adjust the width to match the felt body size, as subjectively perceived. Data collected longitudinally across 3 years showed the presence of slight overestimation of own full body size in earlier age, which decreased during the following years. This evidence confirms greater body distortions in earlier childhood, which however decreased, and then stabilized in adulthood, in line with our findings.

The observed perceptual distortions of the own body-parts size, which is in line with other studies in human adults (e.g.^30,31^), may represent a compensation mechanism due to the over-representation of the hand in S1. In primary somatosensory and motor areas, the representation of the hand is magnified (the so-called cortical homunculus), at variance of other body-parts (e.g. legs); such cortical distortions reflect differences in processing motor or sensory functions according to their functional relevance. Perceptual distortion of one’s own body-part size may represent an adaptive way (likely automatic and unconscious) to counteract the distorted cerebral representation of our body and its parts. For instance, body-parts under-represented in the somatosensory cortical maps are paralleled by low tactile spatial sensitivity (e.g.^5,32^), which indicates an over-estimation at the behavioral level. In the same vein, we propose that our findings could be explained by a complementary mechanism: a smaller visual representation of one’s own hand size may compensate the way it is ‘seen’ by the brain from the inside. This strategy could prove useful to optimize the use of our body, regardless of its actual size representation at a neural level.

Our results not only add an important piece of information to the development of body representation, but have clinical implications too. Indeed, distortions and misperceptions of one’s own body-parts constitute a conspicuous feature of numerous serious clinical diseases, including neurological disorders (for example, phantom limbs, somatoparaphrenia, micro- and macro-somatoagnosia), psychiatric conditions (such as eating disorders), but also developmental disorders, such as autism^33^. These clinical conditions are characterized by representational disturbances of body size and shape, which could be modulated in various ways with therapeutic purposes (e.g.^24,34,35^). A future development of the presence study could be the exploration of pathological perceptual distortions of body-parts size in neurodevelopmental disorders, also assessing whether they could impact on sensory and motor symptoms.

Finally, our study presents a main limitation; indeed, we only tested children between age 4 and 6 years of age, which prevented us from drawing more conclusive findings about the developmental trajectory of hand size distortions.

Moreover, future studies should assess whether these perceptual distortions extend to other body-parts in children, as done in adults too (see^5,31^).

In conclusion, our study has provided evidence in favor of perceptual distortions of the representation of own body-parts size that are already in place by age 4, weaken by adulthood, and thus suggest that body distortions may at least in part possess an experience-dependent plastic nature.

## Materials and Methods

### Participants

Ninety healthy children (39 females; 72 right-handed; mean age ± standard deviation = 5.2 ± 6.56 years; range = 4–6 years) and seventy-eight healthy adults (55 females; 72 right-handed; mean age ± standard deviation = 23.48 ± 3.66 years; range = 18–42 years) were recruited. Handedness was assessed by the standard questionnaire^36^; all participants had normal or corrected-to-normal vision. Twelve children did not complete the experiment and were therefore excluded from statistical analyses; hence the final sample comprised 78 children. Children were recruited from two kindergartens around the city of Milan (‘Bruno Munari’ and ‘Via Gallina di Bareggio’, San Martino di Bareggio, Italy). Adults were students recruited at the University of Milano-Bicocca (Italy).

The study was approved by the local Ethical Committee of the University of Milano-Bicocca and conducted in line with the Declaration of Helsinki. Participants were naïve both to the experimental procedure and to the purpose of the study. The parents of the children gave written informed consent prior to child’s testing. Adult participants signed an informed consent before being tested.

### Experimental procedure

**Figure 4:**
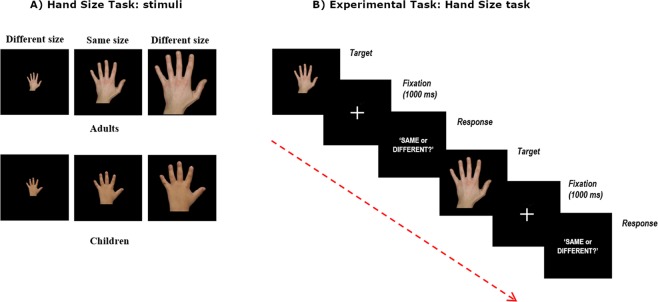
HST (**A**) Stimuli were pictures of the participant’s left and right hands with different sizes, with the respect to the participant’s real hand: 0% (*same* size trial), smaller or bigger by 3%, 6%, 9%, 12% or 15% (*different* size trial). (**B**) The task required to judge whether the size of the viewed hand matched («Same» response) or not («Different» response) the size of their own (out-of-view) hand (2 forced-choice task).

Participants underwent the Hand Size Task (HST^24^), a 2-forced-choice visual perceptual task developed in order to assess the perceptual representation of one’s own hand size. In the HST, stimuli were pictures of the participants’ left hand, taken with a digital camera (ASUS Go 5′′ HD) before starting the task and then presented on a LCD pc screen (Fig. 4a). During the acquisition of each picture, participants were required to insert their own left hand in a wooden box open on its upper side (length = 60 cm, height = 20 cm, width = 30 cm). Pictures were acquired by placing the digital camera above the upper side of the box, thus each picture was taken at the same distance (i.e. 20 cm) for all participants, with the same zoom settings. This procedure allowed to prevent shape distortions into the pictures. Then, the participant’s hand was measured and each hand picture was scaled using the GIMP software considering the pixel resolution of the screen used to run the task, so that pictures reflected the participant’s hand real size. Each picture was presented in egocentric view, at the center of the LCD PC screen on a uniform black background for 1500 ms, followed by a white fixation cross presented at the center of the black screen (see Fig. 4b). Stimuli were scaled in order to be of the same size (i.e., *same size trials*, 0% of hand size variation), smaller (−) or bigger (+) (i.e. *different size trials*: from −15% to +15% in steps of 3%) with respect to the participant’s hand dimension. For each hand size (total = 11), 12 trials were given (total = 132 trials; task duration = ~6 min). For children, the number of stimuli and the procedure were slightly modified in order to facilitate the task for their age. In particular, for each hand size (N = 11), the number of trials presented was 6 (total = 66 trials; task duration = ~15 min). Furthermore, the stimuli were presented in two different blocks (i.e. 33 trails for each block), separated by a 5 minutes interval. Moreover, differently from the adult group, stimuli presentation was not time limited, so that each trial was timed-out by the participant’s response, followed by a white fixation cross presented at the center of the screen.

For both groups, stimuli presentation and randomization were computer-controlled (E-Prime Software, Psychology Software Tools Inc., Pittsburgh, PA), which was used to run the task and to record subject’s responses. During the task, in each trial, the hand size was re-sized on-line by the E-Prime Software. During the HST, participants comfortably sat in an armchair in front of the PC screen at a viewing distance equal to their own forearm, in a dimly-illuminated room; both the left and right hands were hidden under a wooden panel, to prevent direct online size matching. On each trial, participants were presented with the hand stimulus and had to estimate whether it matched («Same» response) or not («Different» response) the size of their own hand. Adults responded with their right hand by pressing the corresponding response button of the PC mouse (i.e. right button for «Same» responses, left button for the «Different» responses). For the children group, in each trial the response button was pressed by the experimenter, after the participant’s verbal response. Before the experimental session, a training session allowed participants to familiarize with the task.

## Data Availability

Data are available from the corresponding author upon request.
